# Variable-Gain Control for Respiratory Systems

**DOI:** 10.1109/TCST.2018.2871002

**Published:** 2018-10-10

**Authors:** Bram Hunnekens, Sjors Kamps, Nathan Van De Wouw

**Affiliations:** 1 DEMCON Macawi Respiratory Systems5692EnschedeThe Netherlands; 2 ASM Laser Separation International (ALSI) B.V.6641BeuningenThe Netherlands; 3 Department of Mechanical EngineeringEindhoven University of Technology5600EindhovenThe Netherlands; 4 Department of Civil, Environmental, and Geo-EngineeringUniversity of MinnesotaMinneapolisMN55455USA; 5 Delft Center for Systems and ControlDelft University of Technology 2860 2628DelftThe Netherlands

**Keywords:** Mechanical ventilation, performance, respiratory systems, variable-gain control

## Abstract

In this paper, we introduce a variable-gain control strategy for mechanical ventilators in the respiratory systems. Respiratory systems assist the patients who have difficulty breathing on their own. For the comfort of the patient, fast pressure buildup (and release) and a stable flow response are desired. However, linear controllers typically need to balance between these conflicting objectives. In order to balance this tradeoff in a more desirable manner, a variable-gain controller is proposed, which switches the controller gain based on the magnitude of the patient flow. The effectiveness of the control strategy is demonstrated in experiments on different test lungs.

## I. Introduction

**Fig. 1.**
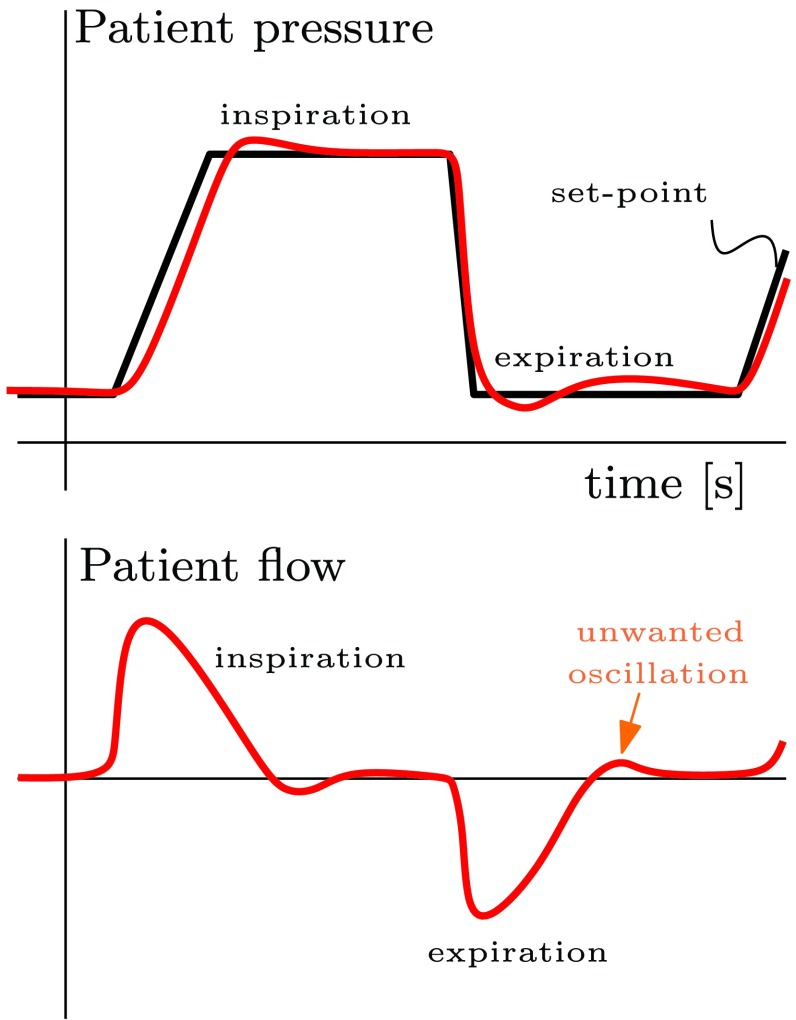
Schematic of a typical breathing cycle during the mechanical ventilation.

Mechanical ventilation is used in hospitals in order to assist the patients who have difficulty breathing on their own. A mechanical ventilator increases the pressure in order to fill the lungs with air during an inspiration, and pressure is decreased in order to release the air from the lungs during an expiration. This is schematically depicted in Fig. 1.

A good ventilator control design ensures that the target pressure is accurately tracked by the ventilator. Moreover, in case a patient can still partly breathe on his/her own, a ventilator should synchronize the controlled pressure profile with the inspiratory and expiratory efforts of the patient, for the comfort and safety of the patient. In other words, if a patient tries to inhale, the ventilator should recognize this (a so-called *trigger*) and support this effort by increasing the pressure. In case a patient tries to exhale, a ventilator should support this effort by decreasing the pressure (such that the patients lungs can passively exhale). This so-called patient-ventilator synchrony is important for the comfort of the patient, as it is clear from several studies in the literature [1], [4], [8], [16], [18], [22], [24]. Asynchrony can lead to prolonged stay in the hospital and even associates with higher mortality [4].

In order to achieve a fast pressure buildup and release during ventilation (accurate tracking), a high-gain pressure controller is preferred. On the other hand, a low-gain pressure controller is preferred in terms of keeping the amount of unwanted oscillations (see Fig. 1) in the patient flow signal small, because this can result in false triggers, which may lead to unwanted induction of an inhalation cycle. Therefore, there is a tradeoff between applying a high-gain controller for accurate pressure tracking and a low-gain controller for obtaining a stable flow response avoiding false inhalation triggers. In order to balance this tradeoff in a more desirable manner, we propose to use a variable-gain control strategy in this paper.

Current mechanical ventilators in hospitals are often highly flexible and versatile machines with many possible ventilation modes [6], [13]. Many ventilation modes synchronize the machine support with the patient effort for the comfort and safety of the patient. Although computer-controlled ventilation is common, a few research studies have been done on the actual controller design, or companies are unwilling to share their knowledge about this subject and, therefore, publications in this area are unfortunately scarce. Some control-related research has been performed in the area of mechanical ventilation focusing on iterative learning control (ILC) [19], funnel-based control [17], or general control applications in ventilation [5], [6], [25]. However, from the experience of the authors, common practice in the existing ventilator systems is that the linear PID-type feedback controllers are used in order to reach the target pressures accurately. Note that the application of accurate feedforward is difficult in mechanical ventilators due to the fact that the patient (lung) dynamics is uncertain, the properties of the hose and bacterial filters attached are typically unknown, and the breathing efforts of the patients are unpredictable. Therefore, the robust PID-type controllers are practically used for a large range of different patient-lung characteristics, which balance between the conflicting tradeoffs of high-gain versus low-gain control. The ILC might be seem as a suitable solution for a repetitive process of mechanical ventilation; however, with the patients triggering breaths, this inherently becomes a nonrepetitive process. Moreover, the “plant” used in the ILC controller incorporates the human patient, which makes it an even more challenging strategy to apply in practice.

As outlined earlier, there exists a tradeoff between the low-gain and the high-gain control in the mechanical ventilation. By using a variable-gain controller, the high-gain controller can only be applied when needed (during pressure buildup and release) and a low-gain controller can be applied when a stable flow response is needed (when the patient flow is small), such that the tradeoff can possibly be balanced in a more desirable manner. The variable-gain control strategy has been used extensively in the area of motion control [3], [11], [12], [15], [23]. In these works, the variable-gain control approach is also used to balance between the conflicting control objectives, i.e., low-gain disturbance suppression versus sensitivity to high-frequency noise (i.e., the waterbed effect [20]) or overshoot versus removing steady-state errors. However, the variable-gain control in the mechanical ventilation is novel and targets the essentially different performance tradeoffs.

Note that in this paper, we focus on the actual low-level pressure control itself but not on the higher level ventilation modes such as proportional pressure support, pressure-controlled volume regulation, tube compensation, and so on (see [5], [7]), which generate a higher level pressure target for the pressure controller. Actually, the low-level variable-gain control technique presented here can be used in combination with these higher level ventilation modes in order to control the airway pressure to the desired pressure targets set by these modes.

The main contribution of this paper can be summarized as follows. First, we introduce a variable-gain control approach that can be applied to mechanical ventilators in order to balance the tradeoff between pressure buildup and a stable flow response in a more desirable manner. Second, we apply the proposed strategy experimentally to a real mechanical ventilator and assess the performance through parameter studies. In these experiments, we assess the stability and performance for a large range of patient-hose combinations in order to illustrate the robustness of the proposed strategy.

The remainder of this paper is organized as follows. In Section II, we present a mathematical model of the respiratory system and simulation results when using a linear control strategy. Section III introduces the variable-gain control strategy and its corresponding stability conditions. In Section IV, we present the experimental results on a real mechanical ventilator. Finally, we present the conclusions and recommendations in Section V.

## II. Linear Control of a Respiratory System

We introduce the basic components of a typical respiratory system and derive a dynamical model of the system in Section II-A. In Section II-B, we illustrate the performance tradeoff arising when using the linear control, which motivates the use of a variable-gain control strategy for mechanical ventilators.

### A. Mathematical Model of a Respiratory System

**Fig. 2.**
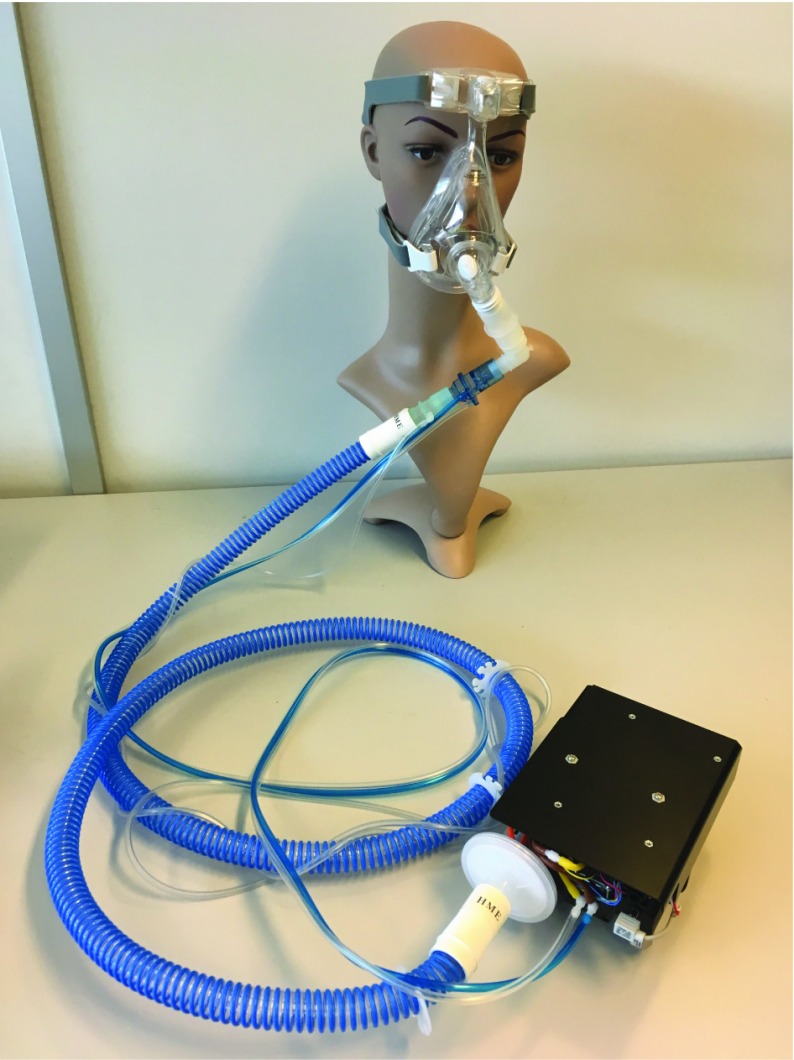
Photograph of a respiratory system with a hose that connects to a patient.

**Fig. 3.**
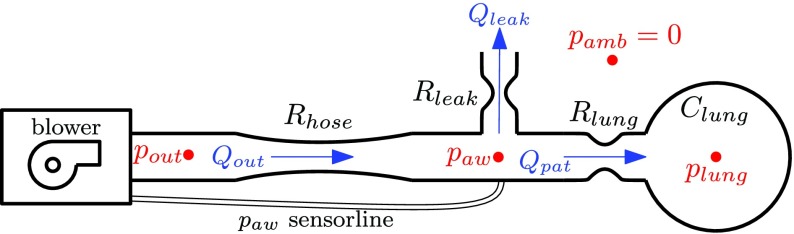
Schematic of a respiratory system showing the different pressures (red), flows (blue), and resistances and compliance (black).

A picture of a respiratory system can be found in Fig. 2. Schematically, this respiratory system can be depicted, as shown in Fig. 3. The system is operated by means of a (centrifugal) blower system, which pressurizes the ambient air in order to ventilate the patient. A hose is used to connect the respiratory module to the patient. The flow $Q_{\mathrm{ out}}$ that leaves the system runs through the hose toward the patient. The patient exhales partly back through the blower and partly through a leak in the hose near the patients mouth (see Fig. 3). The leak, with leak resistance $R_{\mathrm{ leak}}$, is used to refresh the air in the hose in order to ensure that the patient does not inhale his/her own exhaled low-oxygen, CO_2_-rich air.

Using the conservation of flow, the output flow $Q_{\mathrm{ out}}$, patient flow $Q_{\mathrm{ pat}}$, and leakage flow $Q_{\mathrm{ leak}}$ are related as follows:

\begin{equation*} Q_{\mathrm{ pat}} = Q_{\mathrm{ out}} - Q_{\mathrm{ leak}}.\tag{1}\end{equation*}

The pressure at the module outlet is the output pressure $p_{\mathrm{ out}}$. Due to the hose resistance $R_{\mathrm{ hose}}$, the output pressure $p_{\mathrm{ out}}$ is not equal to the so-called airway pressure $p_{\mathrm{ aw}}$ at the patients mouth. This airway pressure $p_{\mathrm{ aw}}$ is the performance variable that is being controlled and measured using a pressure sensor on the module (see Fig. 3). The pressure inside the lungs [with lung compliance (elastance) $C_{\mathrm{ lung}}$ and resistance $R_{\mathrm{ lung}}$] is defined as $p_{\mathrm{ lung}}$ and cannot be measured in general. Assuming linear resistances $R_{\mathrm{ lung}}$, $R_{\mathrm{ leak}}$, and $R_{\mathrm{ hose}}$, the pressure drop across these resistances can be related to the flow through these resistances

\begin{align*} Q_{\mathrm{ out}}=&\frac {p_{\mathrm{ out}}-p_{\mathrm{ aw}}}{R_{\mathrm{ hose}}} \\ Q_{\mathrm{ leak}}=&\frac {p_{\mathrm{ aw}}}{R_{\mathrm{ leak}}} \\ Q_{\mathrm{ pat}}=&\frac {p_{\mathrm{ aw}}-p_{\mathrm{ lung}}}{R_{\mathrm{ lung}}}\tag{2}\end{align*}

and where the lung pressure satisfies the following differential equation:

\begin{equation*} \dot p_{\mathrm{ lung}} = \frac {1}{C_{\mathrm{ lung}}} Q_{\mathrm{ pat}}.\tag{3}\end{equation*}

Note that pressures are all expressed with respect to ambient pressure; therefore, the ambient pressure is considered zero (see Fig. 3).

Combining (2) and (3), the lung dynamics can be written as

\begin{equation*} \dot p_{\mathrm{ lung}} = \frac {p_{\mathrm{ aw}}-p_{\mathrm{ lung}}}{C_{\mathrm{ lung}}R_{\mathrm{ lung}}}\tag{4}\end{equation*}

which is a typical $RC$-system: given an airway pressure $p_{\mathrm{ aw}}$, the lung pressure will reach this airway pressure with a speed related to the characteristic $RC$-time of the system, given by $C_{\mathrm{ lung}}R_{\mathrm{ lung}}$. Substituting and rewriting (2) in (1) result in the following relation for the airway pressure:

\begin{equation*} p_{\mathrm{ aw}} = \frac {\frac {1}{R_{\mathrm{ lung}}}p_{\mathrm{ lung}} + \frac {1}{R_{\mathrm{ hose}}}p_{\mathrm{ out}}}{\frac {1}{R_{\mathrm{ lung}}} + \frac {1}{R_{\mathrm{ hose}}} + \frac {1}{R_{\mathrm{ leak}}}}.\tag{5}\end{equation*}

Substituting this expression for the airway pressure $p_{\mathrm{ aw}}$ into the lung dynamics (4) results in the following differential equation for the lung dynamics:

\begin{equation*} \dot p_{\mathrm{ lung}} = \frac {-\left ({\frac {1}{R_{\mathrm{ hose}}}+\frac {1}{R_{\mathrm{ leak}}}}\right)p_{\mathrm{ lung}} + \frac {1}{R_{\mathrm{ hose}}}p_{\mathrm{ out}}} {R_{\mathrm{ lung}}C_{\mathrm{ lung}}\left ({\frac {1}{R_{\mathrm{ lung}}} + \frac {1}{R_{\mathrm{ hose}}} + \frac {1}{R_{\mathrm{ leak}}}}\right)}.\tag{6}\end{equation*}

Given (2), (5), and (6), the patient and hose system can be written as a linear state-space system with input $p_{\mathrm{ out}}$, outputs $[p_{\mathrm{ aw}},Q_{\mathrm{ pat}}]^{T}$, and state $p_{\mathrm{ lung}}$

\begin{align*} \dot{ p}_{\mathrm{ lung}}=&A_{h} p_{\mathrm{ lung}} + B_{h} p_{\mathrm{ out}} \tag{7}\\ \begin{bmatrix} p_{\mathrm{ aw}} \\ Q_{\mathrm{ pat}} \\ \end{bmatrix}=&{C_{h}} p_{\mathrm{ lung}} + {D_{h}} p_{\mathrm{ out}}\tag{8}\end{align*}

with

\begin{align*} A_{h}=&-\frac {\frac {1}{R_{\mathrm{ hose}}}+\frac {1}{R_{\mathrm{ leak}}}}{R_{\mathrm{ lung}}C_{\mathrm{ lung}} \left({\frac {1}{R_{\mathrm{ lung}}}+\frac {1}{R_{\mathrm{ hose}}}+\frac {1}{R_{\mathrm{ leak}}}}\right)} \\ B_{h}=&\frac {\frac {1}{R_{\mathrm{ hose}}}}{R_{\mathrm{ lung}}C_{\mathrm{ lung}}\left({\frac {1}{R_{\mathrm{ lung}}}+\frac {1}{R_{\mathrm{ hose}}}+\frac {1}{R_{\mathrm{ leak}}}}\right)} \\ C_{h}=&\begin{bmatrix} \frac {\frac {1}{R_{\mathrm{ lung}}}}{\frac {1}{R_{\mathrm{ lung}}}\!+\!\frac {1}{R_{\mathrm{ hose}}}\!+\!\frac {1}{R_{\mathrm{ leak}}}} \!-\!\frac {\frac {1}{R_{\mathrm{ hose}}}+\frac {1}{R_{\mathrm{ leak}}}}{R_{\mathrm{ lung}}\left({\frac {1}{R_{\mathrm{ lung}}}+\frac {1}{R_{\mathrm{ hose}}}+\frac {1}{R_{\mathrm{ leak}}}}\right)} \end{bmatrix}^{T}\!\! \\ D_{h}=&\begin{bmatrix} \frac {\frac {1}{R_{\mathrm{ hose}}}}{\frac {1}{R_{\mathrm{ lung}}}+\frac {1}{R_{\mathrm{ hose}}}+\frac {1}{R_{\mathrm{ leak}}}} \frac {\frac {1}{R_{\mathrm{ hose}}}}{R_{\mathrm{ lung}}\left({\frac {1}{R_{\mathrm{ lung}}}+\frac {1}{R_{\mathrm{ hose}}}+\frac {1}{R_{\mathrm{ leak}}}}\right)} \end{bmatrix}^{T} \\\tag{9}\end{align*}

or, equivalently, in transfer function notation

\begin{equation*} H(s) = C_{h}(sI-A_{h})^{-1}B_{h} + D_{h}.\tag{10}\end{equation*}

**Fig. 4.**
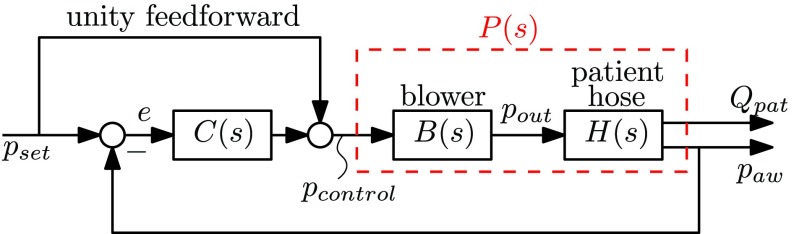
Closed-loop control scheme with a linear controller $C(s)$.

The module output pressure $p_{\mathrm{ out}}$ can accurately be generated by the blower system. The characteristics of the blower have been identified in terms of a steady-state characteristic, which ideally makes the mapping between the output pressure target $p_{\mathrm{ control}}$ (see Fig. 4) and the actual output pressure $p_{\mathrm{ out}}$ that is equal to 1. However, the blower is a dynamical system with inertia; therefore, the actual system has roll-off for high frequencies, which can be modeled as a second-order low-pass filter

\begin{equation*} B(s)=\frac {p_{\mathrm{ out}}(s)}{p_{\mathrm{ control}}(s)}=\frac {\omega _{n}^{2}}{s^{2}+2 \zeta \omega _{n} s + \omega ^{2}_{n}} \tag{11}\end{equation*}

with $\omega _{n} = 2\pi 30$ and damping ratio $\zeta = 1$, corresponding to an actual experimental blower. In the state-space format, (11) can be written as

\begin{align*} \dot {x}_{b}=&A_{b} x_{b} + B_{b} p_{\mathrm{ control}} \\ p_{\mathrm{ out}}=&C_{b} x_{b} \tag{12}\end{align*}

with state $x_{b}~\in \mathbb {R}^{2}$, output $p_{\mathrm{ out}}$, and control input $p_{\mathrm{ control}}$, and system matrices

\begin{align*} A_{b}=&\begin{pmatrix} -2 \zeta \omega _{n} &\quad -\omega ^{2}_{n} \\ 1 &\quad 0 \end{pmatrix} \quad B_{b} = \begin{pmatrix} 1 \\ 0 \end{pmatrix} \\ C_{b}=&\big (0 \quad \omega _{n}^{2} \big).\tag{13}\end{align*}

By coupling the patient hose system dynamics (7) and the blower dynamics (12), the general state-space form of the plant $P(s)$ (to be controlled by the feedback controller; see Fig. 4) can be formulated as

\begin{align*} \dot {x}_{p}=&\begin{bmatrix} \dot {x}_{b} \\ \dot {p}_{\mathrm{ lung}} \end{bmatrix} = \underbrace {\begin{bmatrix} A_{b} &\quad 0 \\ B_{h} C_{b} &\quad A_{h} \end{bmatrix}}_{A_{p}} \begin{bmatrix} x_{b} \\ p_{\mathrm{ lung}} \end{bmatrix} + \underbrace {\begin{bmatrix} B_{b} \\ 0 \end{bmatrix}}_{B_{p}}p_{\mathrm{ control}} \\ z=&\begin{bmatrix} p_{\mathrm{ aw}} \\ Q_{\mathrm{ pat}} \end{bmatrix} = \underbrace {[D_{h} C_{b} \quad C_{h}]}_{C_{p}} \begin{bmatrix} x_{b} \\ p_{\mathrm{ lung}} \end{bmatrix} \tag{14}\end{align*}

with transfer function

\begin{equation*} P(s):= \begin{bmatrix} P_{p}(s) \\ P_{Q}(s) \end{bmatrix} = B(s)H(s) = C_{p}(sI-A_{p})^{-1}B_{p}.\qquad \tag{15}\end{equation*}

### B. Performance Tradeoff Using Linear Control

In Section II-A, a mathematical formulation of the plant model $P(s)$ has been derived. In this section, a linear controller $C(s)$ is designed for the system, and the tradeoff between the low-gain control and the high-gain control is illustrated by means of a simulation study.

Implementing the controller results in the closed-loop system where the airway pressure $p_{\mathrm{ aw}}$ is the variable to be controlled (e.g., to track the target ventilation set point $p_{\mathrm{ set}}$), as shown in Fig. 4. The unity feedforward in combination with the identified blower characteristic ensures that the output pressure $p_{\mathrm{ out}}$ can reasonably accurately track the target pressure $p_{\mathrm{ set}}$ by feedforward alone. However, the feedback controller has to compensate for the pressure drop $\Delta p = p_{\mathrm{ out}} - p_{\mathrm{ aw}}$ along the hose and for the variations across the blower characteristics. Note that the pressure drop along the hose is difficult to predict due to several factors as follows.

1) The type of lung attached (i.e., the patient) is in principal unknown. Although the pressure target is *a priori* known, the amount of flow entering a lung depends on the lung resistance $R_{\mathrm{ lung}}$ and the lung compliance $C_{\mathrm{ lung}}$ and is therefore unknown. Hence, because the exact hose system attached is also unknown, the pressure drop along the hose is unknown.
2) During (noninvasive) ventilation, there can be leakage around the mask, which cannot be predicted, and, therefore, also results in an unknown pressure drop.
3) In addition, patients can have spontaneous activity (resulting in a flow and, hence, a pressure drop along the hose), which also cannot be predicted *a priori*.

Therefore, the feedforward control cannot be used to compensate for these effects, and the feedback control needs to be employed.

**Fig. 5.**
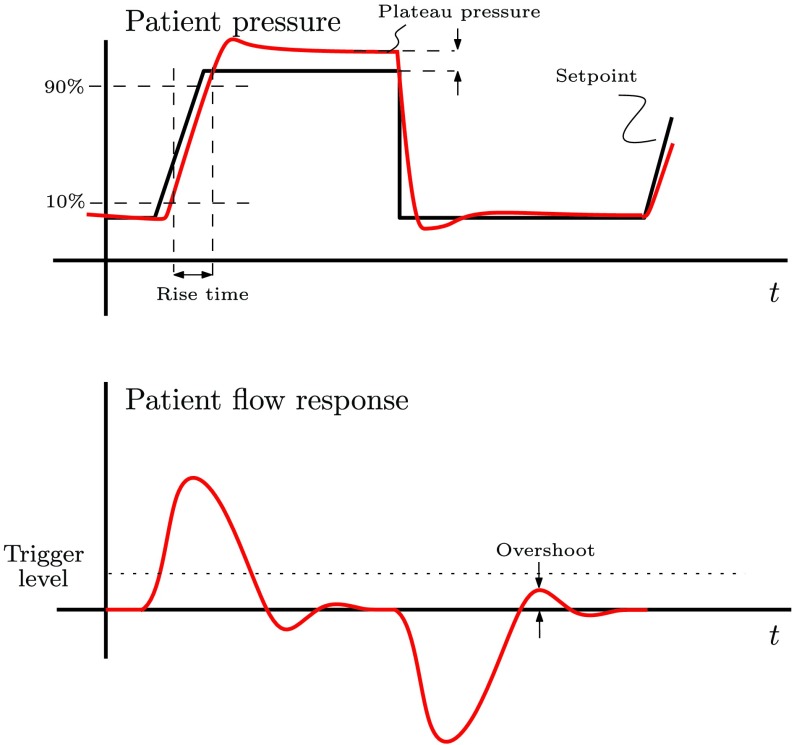
Performance indicators used to evaluate the performance of the respiratory system.

The goal of the pressure controller is to achieve sufficiently fast pressure buildup and accurate tracking of the desired pressure profile $p_{\mathrm{ set}}$ while simultaneously not introducing the oscillations in the flow signal, which may result in false triggers of the inhalation cycle. Quantitatively, these specifications can be formulated as follows (see Fig. 5).

1) The rise time from 10% to 90% of a pressure set point should be approximately 200 ms.
2) The pressure at the end of an inspiration, the so-called plateau pressure, should be within a pressure band of ±2 mbar of the pressure set point.
3) The overshoot in the flow during an expiration should be below the triggering value set by the clinician, and a typical value is 2 L/min.

**Fig. 6.**
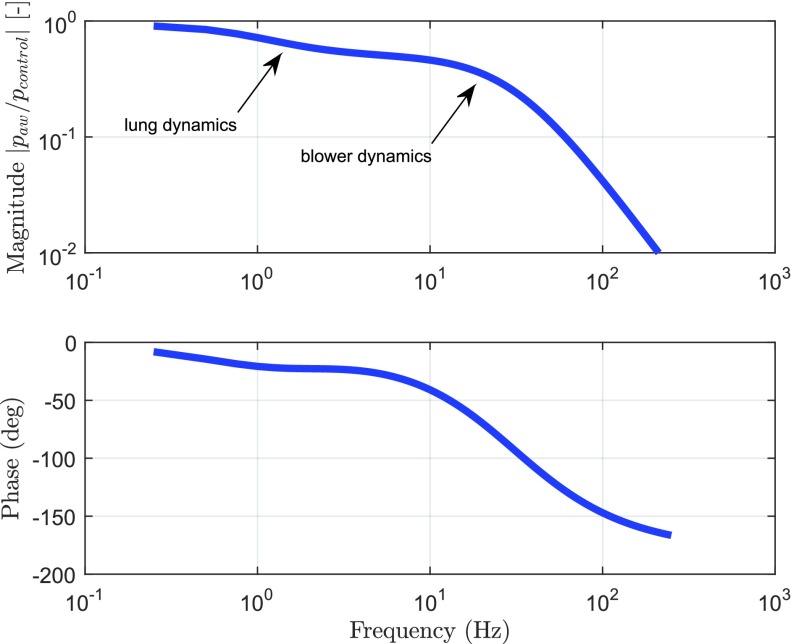
Bode plot of the plant (15) to be controlled. The used parameter values are indicated in Table I.

Given the model parameters in Table I, the Bode plot of the plant is shown in Fig. 6. The Bode plot clearly shows the dynamics related to the lung and the blower dynamics. Moreover, note that $P(\omega = 0) < 1$ due to the pressure drop along the hose. In other words, given a constant pressure $p_{\mathrm{ control}}$, there will be a leakage flow $Q_{\mathrm{ leak}}$ through the leakage hole in the hose, which results in a pressure drop $\Delta p$ along the hose, such that $p_{\mathrm{ aw}}/p_{\mathrm{ control}} < 1$ in steady state.

**TABLE I:** Parameters Settings Used for Simulations

Variable	Value	Unit
$R_{\mathrm{ lung}}$	$\frac {5}{1000}$	$\frac {mbar}{\frac {mL}{s}}$
$C_{\mathrm{ lung}}$	20	$\frac {mL}{mbar}$
$R_{\mathrm{ leak}}$	$\frac {60}{1000}$	$\frac {mbar}{\frac {mL}{s}}$
$R_{\mathrm{ hose}}$	$\frac {4.5}{1000}$	$\frac {mbar}{\frac {mL}{s}}$
$\omega _{n}$	$2 \pi 30$	$\frac {rad}{s}$

To cope with such leakage-induced disturbances, the following linear integral feedback controller has been designed:

\begin{equation*} C(s) = \frac {k_{i}}{s}.\tag{16}\end{equation*}

Note that this is a suitable controller type for this system, since it results in low-frequency disturbance suppression, high-frequency roll-off, and a stabilizing −1 slope across the bandwidth of the system.

Remark 1: One could argue to add proportional action to the controller [i.e., $C(s) = k_{i}/s + k_{p}$], but for this plant, it will unnecessarily result in less roll-off for frequencies above the bandwidth and is, hence, considered undesirable.

Remark 2: Although the expiration of a patient lung is a passive process, a certain amount of control action is needed in order to achieve the set expiration pressure level. Especially, in the system under consideration, there is always an intentional leak (see Fig. 3), and hence, a certain amount of control action is needed in order to maintain the set expiration pressure level.

**Fig. 7.**
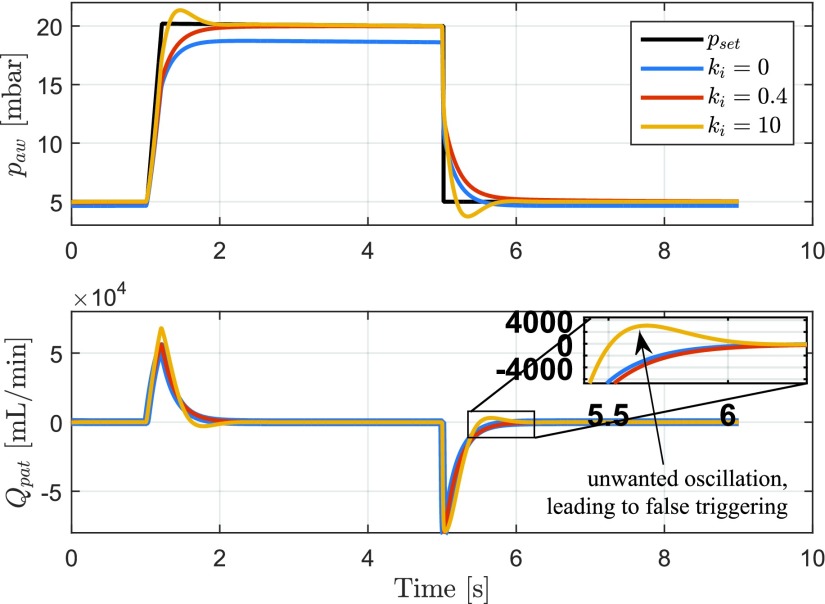
Simulation result of the closed-loop system using no controller, a low-gain controller ($k_{i} = 0.4$), and a high-gain controller ($k_{i} = 10$).

Three different simulation results are shown in Fig. 7.

1) Open-loop control, i.e., using $C(s) = k_{i} = 0$. This simulation result shows the need for a feedback controller in order to achieve the target pressure accurately.
2) A low-gain integral feedback controller $C(s)$ as in (16) and $k_{i} = 0.4$. The low-gain controller does achieve the target pressure albeit slowly, but it induces a flow response without overshoot (favorable to avoid false inspiration triggers).
3) A high-gain integral feedback controller $C(s)$ as in (16) and with $k_{i} = 10$. The high-gain controller achieves the target pressure quickly, but it induces an unwanted oscillation in the patient flow, which may result in false patient flow triggering.

In order to balance this tradeoff between the high-gain control and the low-gain control in an improved manner, we propose the use of a variable-gain controller in Section III.

## III. Variable-Gain Control of a Respiratory System

### A. Variable-Gain Control Scheme

**Fig. 8.**
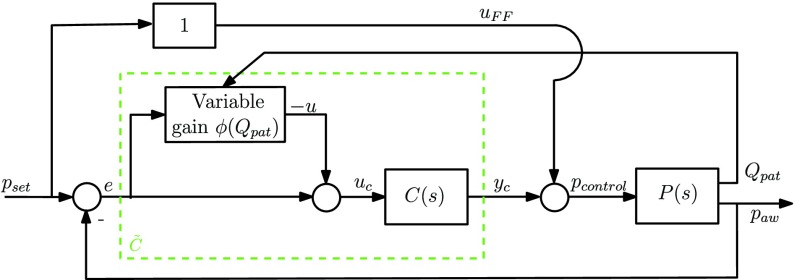
Schematic of the variable-gain control scheme, where the variable-gain controller is denoted by $\tilde C$.

In the proposed variable-gain control scheme, the controller switches between a high-gain controller and a low-gain controller in order to balance the tradeoff between a fast pressure performance and having a steady flow response without inducing false inspiration triggers. The variable-gain controller $\tilde C$ consists of a linear controller $C(s)$ and a variable-gain element $\varphi = \phi e$, with $e = p_{\mathrm{ set}}-p_{\mathrm{ aw}}$, as shown in Fig. 8.

**Fig. 9.**
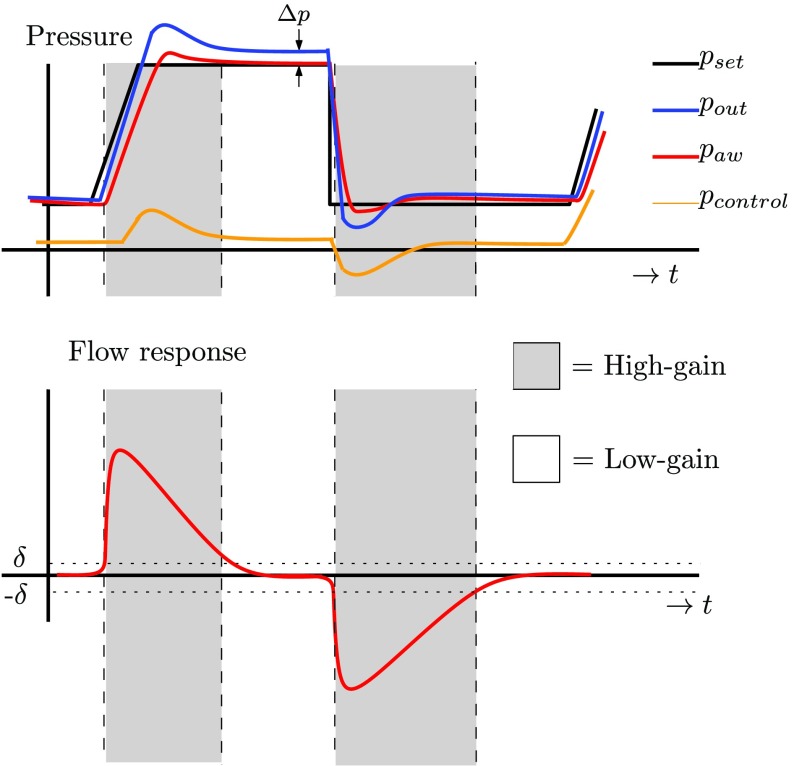
Typical ventilation pattern with the desired intervals of high- and low-gain control.

A relevant question is: how to design a switching law between low- and high-gain control settings to be encoded in the design of the gain $\phi $? Consider a typical ventilation cycle in Fig. 9, which indicates a typical output pressure $p_{\mathrm{ out}}$ (at the module), a patient airway pressure $p_{\mathrm{ aw}}$ (at the patient), and a typical control effort $p_{\mathrm{ control}}$ which is needed to overcome the pressure drop along the hose. When the patient flow $Q_{\mathrm{ pat}}$ is small, the pressure drop $\Delta p = p_{\mathrm{ aw}}-p_{\mathrm{ out}}$ along the hose is almost constant, and the low-gain controller is favorable, since this minimizes the oscillations in the flow response. For large flows, however, during pressure buildup and pressure release, a higher controller gain is desired (see Fig. 9) in order to compensate for the pressure drop along the hose quickly. Therefore, we propose to switch the controller gain based on the patient flow $Q_{\mathrm{ pat}}$

\begin{equation*} \varphi (e,Q_{\mathrm{ pat}}) = \phi (Q_{\mathrm{ pat}}) e\tag{17}\end{equation*}

in which $\phi (Q_{\mathrm{ pat}})$ should be designed to be large for large flows (high-gain) and small for small flows (low gain). Schematically, this is shown in Fig. 9. A specific choice for the nonlinear gain can be a “switch” nonlinearity

\begin{equation*} \phi (Q_{\mathrm{ pat}}) = \begin{cases} 0, & \text {if}~|Q_{\mathrm{ pat}}| \leq \delta \\ \alpha, & \text {if}~|Q_{\mathrm{ pat}} | > \delta \end{cases} \tag{18}\end{equation*}

with $\delta $ the switching length and $\alpha $ the additional gain. Note that the nonlinearity $\varphi (e,Q_{\mathrm{ pat}})$ satisfies a certain sector condition (see [14]): it holds that $\varphi \in [0,\alpha]$ for all $e,Q_{\mathrm{ pat}}~\in ~\mathbb {R}$ and holds irrespective of the switch length $\delta $. Therefore, $\delta $ is purely a performance-based variable and stability invariant. On the other hand, the design proposed here differs essentially from variable/gain designs common in the literature [11], [23], where the variable gain ($\phi $) typically depends on the feedback variable ($e$), while in the design in (18), it depends on another measured quantity ($Q_{\mathrm{ pat}}$).

Remark 3: In this paper, we consider the pressure-controlled ventilation modes, where the pressure is the variable to be controlled during inspiration and expiration. In the so-called flow-controlled modes, the flow is controlled during inspiration, and the pressure is controlled during expiration. In such flow-controlled modes, the proposed variable-gain control scheme can still be used for the pressure-controlled expiration phase.

### B. Stability of the Variable-Gain Control Scheme

**Fig. 10.**
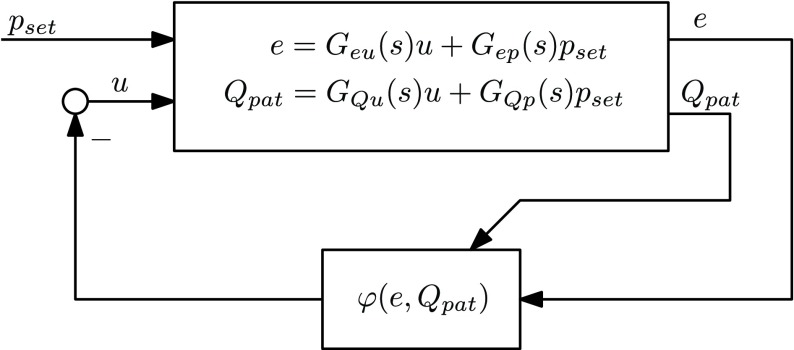
Linear closed-loop system with a static nonlinearity in the feedback loop, in the literature referred to as a Lur’e form.

The resulting closed-loop system can be represented as a feedback connection of a linear dynamical system and a static nonlinearity in the feedback loop, i.e., as a Lur’e-type system [14] (see Fig. 10) of the following state-space form:

\begin{align*} \dot x=&Ax + B p_{\mathrm{ set}} + B_{u} u \\ \begin{bmatrix} e \\ Q_{\mathrm{ pat}} \end{bmatrix}=&C x + D p_{\mathrm{ set}} \tag{19}\\ u=&- \varphi (e,Q_{\mathrm{ pat}}) = \phi (Q_{\mathrm{ pat}}) e.\end{align*}

The relevant transfer functions of the Lur’e-type system associated with the output $e$ are given by

\begin{align*} e=&p_{\mathrm{ set}} - p_{\mathrm{ aw}} = \frac {P_{p}(s)C(s)}{1+P_{p}(s)C(s)} u + \frac {1-P_{p}(s)}{1+P_{p}(s)C(s)} p_{\mathrm{ set}} \\=:&G_{eu}(s) u + G_{\text {ep}}(s) p_{\mathrm{ set}}\tag{20}\end{align*}

where we used the definition of $P_{p}(s)$ as in (15). Note that $G_{eu}(s)$ represents the complementary sensitivity function.

For a linear system, stability, which can be assessed through the Nyquist criterion, guarantees a bounded state response under bounded inputs acting on the system. For a nonlinear system, this property of a bounded state response under bounded inputs is not trivial and is captured by the notion of input-to-state stability (ISS) [21]. The following theorem provides the sufficient conditions under which the closed-loop variable-gain control system (19) is ISS with respect to the input $p_{\mathrm{ set}}$ (and hence results in bounded pressures under the influence of the bounded input $p_{\mathrm{ set}}$):

Theorem 1: Consider the closed-loop variable-gain control system in (19). Suppose the following holds.

1) The transfer function $1+\alpha G_{eu}(s)$ is strictly positive real.
  (a) $G_{eu}(s)$ is Hurwitz.
  (b) *Re* $(G_{eu}(j\omega)) > -({1}/{\alpha })$ for all $\omega \in \mathbb {R}$.
  (c) $1+\alpha G_{eu}(\infty) > 0$.
2) The nonlinearity $\varphi (e,Q_{\mathrm{ pat}})$ satisfies the $[0,\alpha]$ sector condition
  \begin{equation*} 0 \leq \frac {\varphi (e,Q_{\mathrm{ pat}})}{e} = \phi (Q_{\mathrm{ pat}}) \leq \alpha\tag{21}\end{equation*}
  for all $e\in \mathbb {R}\setminus \{0\}$, $Q_{\mathrm{ pat}} \in \mathbb {R}$.

Then, the system is ISS with respect to the input $p_{\mathrm{ set}}$.

Proof: The proof follows from circle-criterion-type arguments [2], [10], [14], [26]. In the proofs in these references, typically, the nonlinearity $\varphi (e) = \phi (e) e$ only depends on the error $e$. However, even though the nonlinearity considered here depends on an additional output variable $Q_{\mathrm{ pat}}$ [see (17)], the same proof applies. To see this, note that the nonlinearity $\varphi (e,Q_{\mathrm{ pat}})$ satisfies the sector condition (21) for all $e \in \mathbb {R}$, $e \neq 0$, but also for all $Q_{\mathrm{ pat}} \in \mathbb {R}$, such that a proof along the lines of [10], [14], and [26] can be followed.

Remark 4: Note that the conditions of Theorem 1 can easily be checked. Condition 1a) is satisfied by a proper stabilizing design of the low-gain controller $C(s)$ (which can be checked in the frequency domain using the Nyquist stability theorem [9]). Condition 1b) can be checked in the frequency domain as well by investigating the Nyquist plot of $G_{eu}(s)$. Condition 1c) is usually automatically satisfied due to the fact that $G_{eu}(s)$ has roll off for high frequencies such that $1+\alpha G_{eu}(\infty) = 1 > 0$. Condition 2) can be satisfied by the design of the nonlinearity itself.

In this section, the variable-gain control strategy has been presented, together with the conditions for ISS. In Section IV, we apply and compare linear and variable-gain control by implementation on the experimental respiratory system.

## IV. Experimental Application of Variable-Gain Control

### A. Experimental Results

**Fig. 11.**
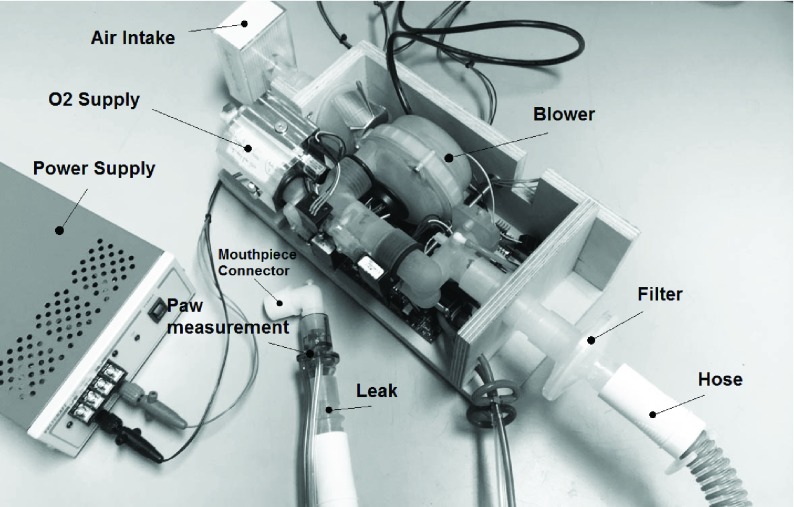
Photograph of the experimental setup highlighting the most important elements of the setup

A picture of the experimental setup can be found in Fig. 11. The most important elements of the setup are highlighted in this figure, such as the blower system, the patient hose, the leakage, and the patient pressure/flow measurement.

**Fig. 12.**
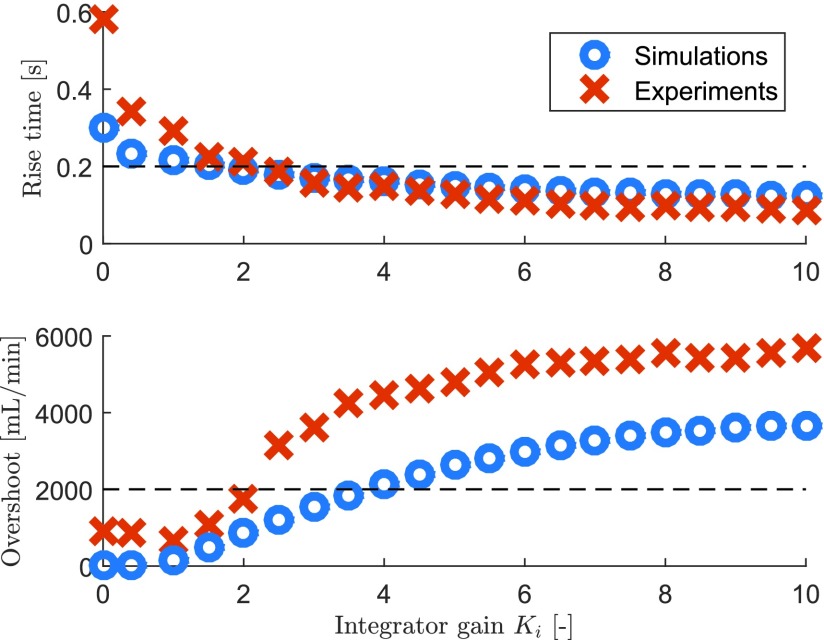
Simulation and experimental results of the rise time and the overshoot for a range of linear controllers as a function of the integrator gain $k_{i}$. Dashed lines indicate the performance specifications as in Fig. 5.

In order to illustrate the tradeoff that linear controllers face when controlling a respiratory module in experiments, consider the rise time and overshoot performance indicators from Fig. 5, which are shown in Fig. 12 for a range of integrator gains $k_{i}$. Clearly, the tradeoff between fast pressure buildup (reflected by the rise time) and overshoot in the flow response can also be observed in experiments. Although qualitatively the results between simulations and experiments match, quantitatively there are some differences. The main reason why the experimental results do not exactly match with the simulations is that the lung resistance $R_{\mathrm{ lung}}$ used in the mechanical test lung is a quadratic resistance (a simple hole), opposed to a linear resistance in simulations. An actual human lung will likely behave more as a linear resistance or a combination of a linear and quadratic resistance.

Note that from Fig. 12, it can be concluded that no integrator gain $k_{i}$ exists that meets both the rise time specification of 0.2 s and the overshoot specification of 2000 mL/min in experiments. Choosing $k_{i} = 2$ would result in a quite good compromise, but it is not a robust setting, considering the fact that a slightly larger $k_{i}$ value already results in a significantly larger overshoot, and a slightly smaller $k_{i}$ value already results in a significantly larger rise time.

In order to balance this tradeoff in a more desirable manner, we will design a variable-gain controller, which should satisfy the ISS stability conditions from Theorem 1.

**Fig. 13.**
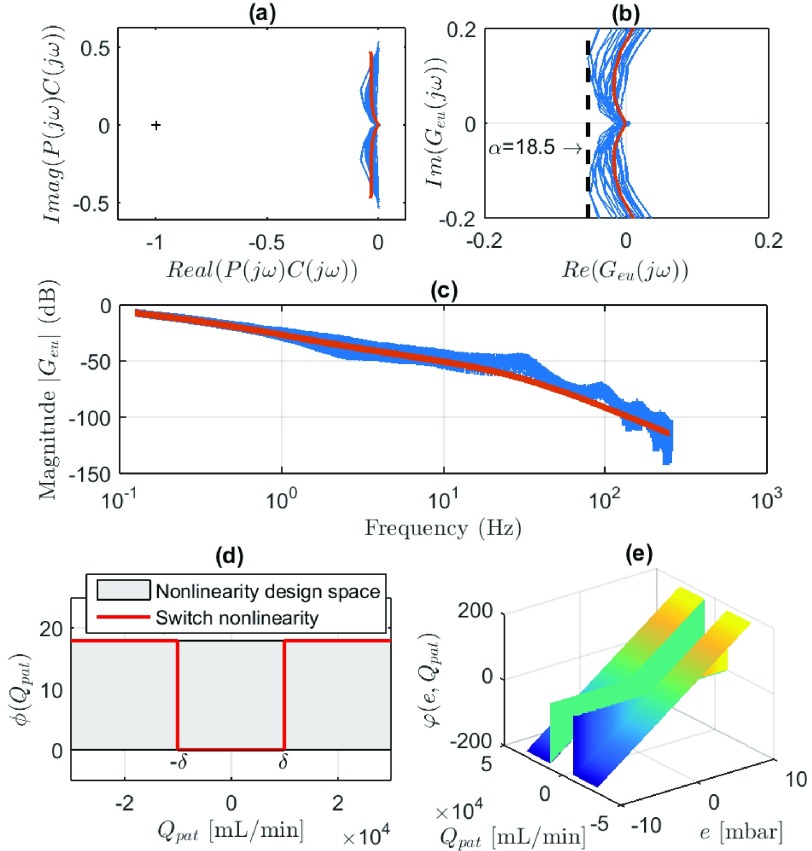
ISS stability conditions. (a) Nyquist plot of open loop $P(s)C(s)$. (b) Nyquist plot of circle-criterion condition on $G_{eu}(s)$. (c) Bode plot of $G_{eu}(j\omega)$ showing roll-off for $\omega \rightarrow \infty $. (d) Switch nonlinearity gain $\phi (Q_{\mathrm{ pat}})$. (e) Output of the nonlinearity $\varphi (e,Q_{\mathrm{ pat}})$ as in (17) and (18).

1) The transfer function $1+\alpha G_{eu}(s)$ is strictly positive real.
  (a) $G_{eu}(s)$ *Must Be Hurwitz:* The low-gain controller is designed to be $C(s) = k_{i}/s = 0.4/s$ [we explicitly choose $k_{i}$ to be small since this results in a stable flow response (see Fig. 12), while the additional gain will only be added when needed, by means of the variable-gain element]. Note that $G_{eu}(s) = ({P(s)C(s)}/({1+P(s)C(s)}))$ is the complementary sensitivity. Stability of $G_{eu}(s)$ can be investigated by checking the Nyquist criterion for the open-loop $P(s)C(s)$ transfer function. Fig. 13(a) shows the open-loop Nyquist plot for a large range of patient-hose combinations (i.e., many different lungs), as well as for the derived hose model. From this figure, it is clear that condition 1a) is met.
  (b) Re $(G_{eu}(j\omega)) > -({1}/{\alpha })$ *for All* $\omega \in \mathbb {R}$ *:* This condition can also be checked graphically by plotting the Nyquist diagram of $G_{eu}(j\omega)$ [see Fig. 13(b)]. Again, for a large range of possible patient-hose combinations, the condition can be checked. From this, it follows that the maximum additional gain $\alpha = 18.5$^1^ (see [11], [12], [23]).
  (c) $1+\alpha G_{eu}(\infty) > 0$ *:* $G_{eu}(j\omega)$ has roll off due to the low-pass nature of the transfer function of the blower $B(s)$ [see (11) and Fig. 13(c)], such that $1+\alpha G_{eu}(\infty) = 1 > 0$. $\varphi (e,Q_{\mathrm{ pat}})$
2) satisfies the $[0,\alpha]$ sector for all $e$, $Q_{\mathrm{ pat}} \in \mathbb {R}$. By using the designed nonlinearity as in (17) and restricting $\alpha $ to its maximum value of 18.5, this condition is met [see also Fig. 13(d) and (e)].

^1^If additional freedom is desired in the tuning of the maximum gain $\alpha $, one can place an additional filter $F(s)$ in series with the nonlinearity $\varphi (e,Q_{\mathrm{ pat}})$. We do not exploit this freedom here, because it is challenging to design a robust filter $F(s)$ due to the large variability in the patient-hose combinations.

Since both the conditions are satisfied, it can be concluded that the closed-loop system is input-to-state stable for $\alpha < 18.5$. Again, note that this result holds, independent of the tuning of the switching length $\delta $.

**Fig. 14.**
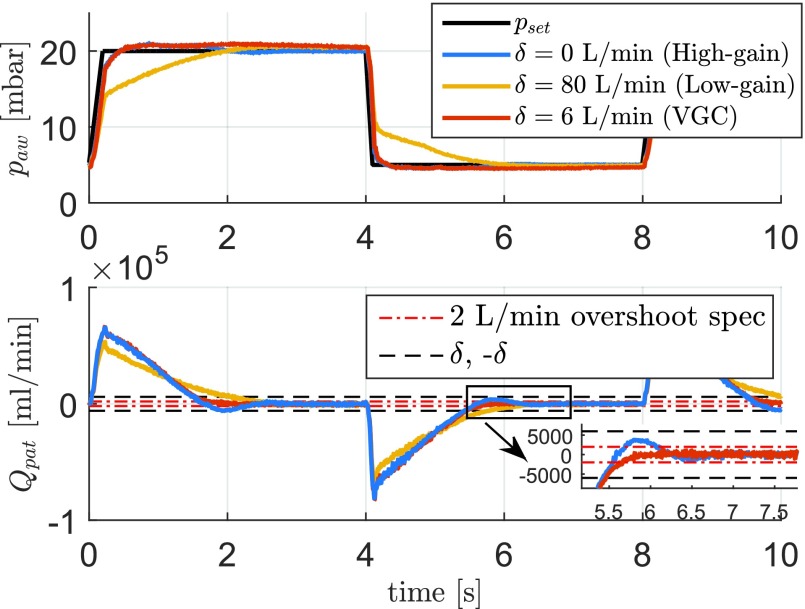
Experimental time-domain response of the linear controllers and a variable-gain controller with $\delta = 6$ L/min. Note that $\delta = 0$ represents the linear high-gain controller and $\delta = 80$ L/min represents the linear low-gain controller.

In order to assess the performance of the variable-gain control strategy in the time domain, consider the experimental results shown in Fig. 14 (for one of the FRFs in Fig. 13). Clearly, the (linear) high-gain controller [corresponding to $\delta = 0$ L/min and $\tilde C = C(s)(1+\alpha)$] has a fast pressure buildup (and pressure release) performance. However, in terms of overshoot in the flow response, the high-gain controller does not perform well. The overshoot exceeds the flow trigger threshold of 2 L/min and would induce the false patient triggers. The low-gain controller, on the other hand, has a stable flow response, but it is too slow in the pressure buildup (and pressure release) in order to meet the desired rise-time performance specification.

The variable-gain controller uses the nonlinearity to differentiate between low/high gains based on the patient flow information provided by the flow sensors. If the patient flow is larger than $\delta $ ($\delta = 6$ L/min in Fig. 14), the additional gain $\alpha $ is applied in order to compensate for the pressure drop along the hose and quickly reach the desired pressure target, similarly as the high-gain controller. However, once the patient lung becomes full, and the patient flow $Q_{\mathrm{ pat}}$ becomes close to zero (and, hence, the pressure drop along the hose is approximately constant), the low-gain controller is used in order to reach a stable flow response without overshoot (see Fig. 14). In this way, the variable-gain controller combines the best of both worlds: trigger levels can be set to a small level, to allow for best possible patient comfort, while still achieving sufficiently fast pressure buildup.

Remark 5: The variable-gain controller limits the amount of patient-flow overshoot by switching to the low-gain controller. Note that this results in the fact that the settling of the variable-gain controller to the end pressure [see in Fig. 14 (top)] takes longer, and however, this is no problem for the application, since the pressure already resides well within the target pressure band of 2 mbar (see specification 2) in Section II-B).

### B. Influence of the Switching Length $\delta$

In this section, we will study the influence of the switching length $\delta $ on the performance and analyze the robustness of the tuning of $\delta $ with respect to plant variations.

**Fig. 15.**
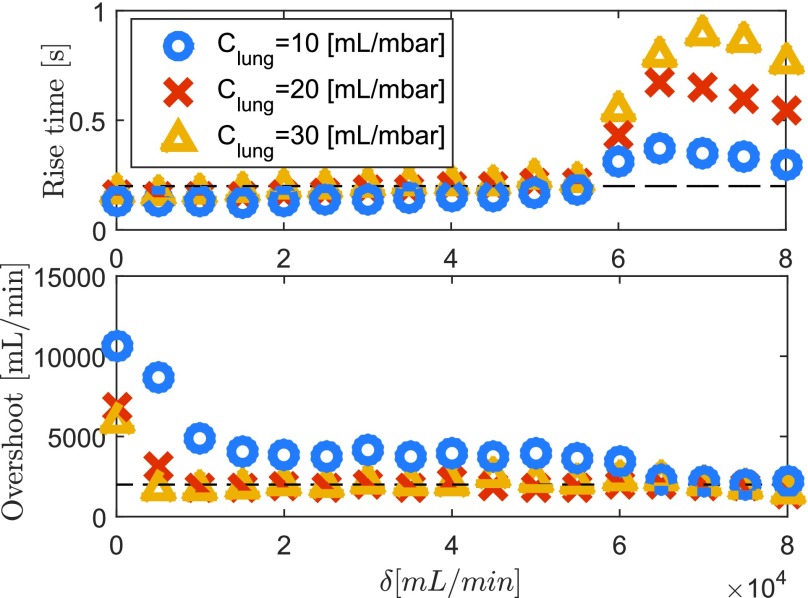
Rise time and overshoot for different lungs as a function of the switching length $\delta $.

In order to study the influence of the switching length $\delta $ on the performance of the variable-gain controller in more detail, consider the results in Fig. 15. Clearly, a smaller $\delta $ value results in a faster rise time, because the additional gain is active for a longer period of time. However, it results in a larger overshoot. The linear controllers ($\delta = 0$ and $\delta = 80$ L/min) need to balance this tradeoff. The variable-gain controller can balance this tradeoff in a more desirable manner, by choosing, for example, $\delta \approx 10$ L/min, which results in a response with a good rise time and also a small overshoot in the flow. Also, note that the region in which overshoot and rise time are both small is quite large (e.g., any $\delta $ between 5 and 30 L/min would perform well), which means that the design is robust with respect to the switching length $\delta $.

In addition, consider the results for the different lung compliances in Fig. 15. Even though the lungs differ significantly, any switching length $\delta $ between 5 and 30 works well for all three different lungs. This illustrates the robustness of the tuning of $\delta $ with respect to different lung characteristics.

## V. Conclusion

The main contribution of this paper is, first, the introduction of a variable-gain control strategy for a mechanical ventilator in order to balance the tradeoff between accurate and fast pressure buildup and a stable flow response in a more desirable manner. Second, the experimental results confirm that indeed a variable-gain controller, which switches gains on the basis of the magnitude of the patient-flow, can outperform its linear counterparts. As a benefit for the patient, trigger levels can be set to a small level, to allow for best possible machine-patient synchronization for the comfort of the patient, while still achieving sufficiently fast pressure buildup that guarantees good breathing support.
